# Supplementary figures and images for: Therapeutic effects of PDGF-AB/BB against cellular senescence in human intervertebral disc
Source: bioRxiv. 2025 Mar 27:2024.10.11.617862. Originally published 2024 Oct 13. Preprint. [Version 2] doi: 10.1101/2024.10.11.617862 (PMC11482872; doi:10.1101/2024.10.11.617862)

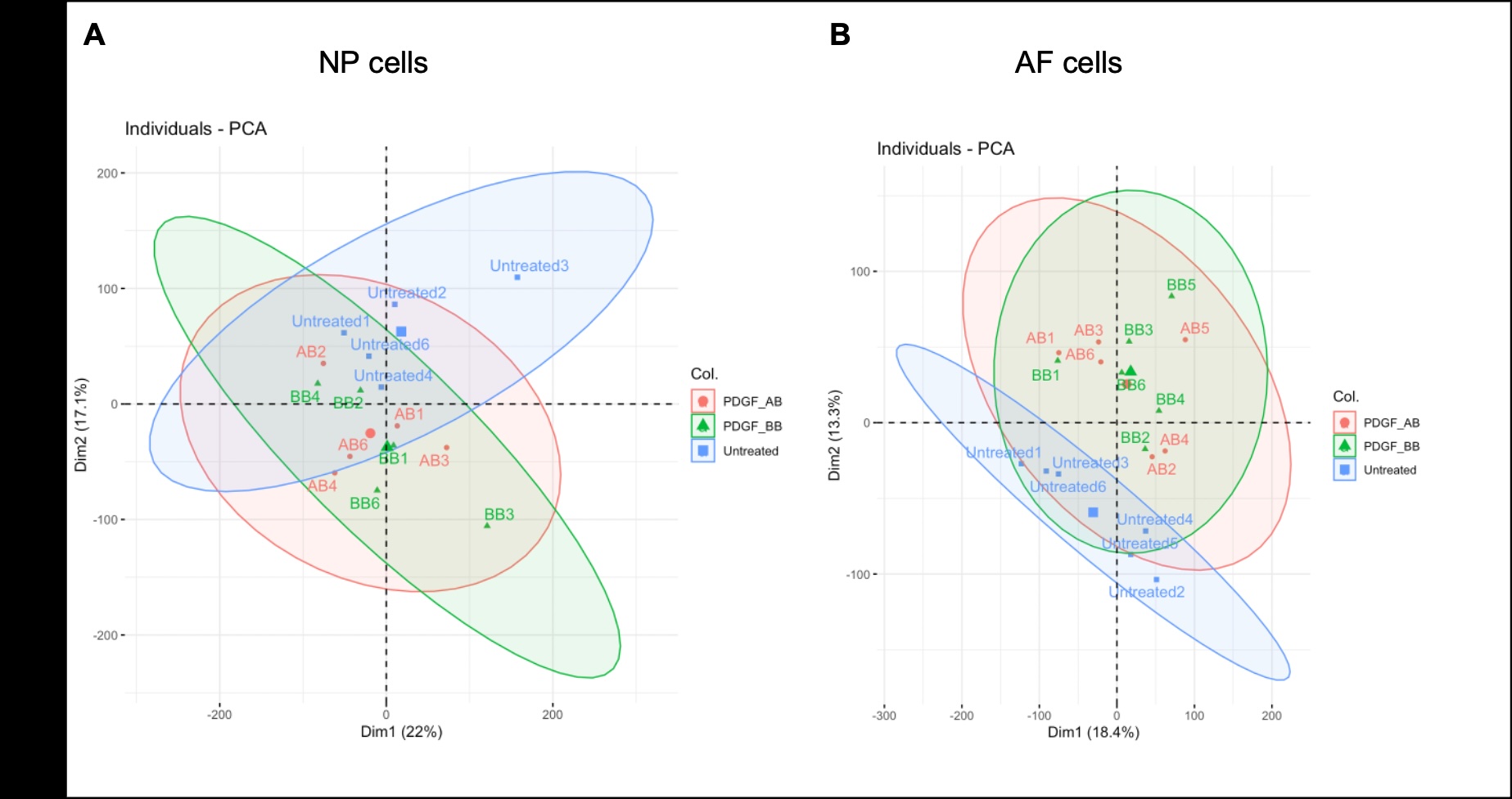

Supplement: Supplement 1 — Supplementary figure 1: Principle component analysis (PCA) of NP and AF samples treated with rhPDGF-AB and BB. A+B) PCA plot of NP (A) cells and AF cell (B) showed the distinct cluster between untreated and treated samples. The clusters of PDGF-AB and BB samples were overlapping. NP: n = 5 each group. AF: n = 6 each group. [file media-1.jpg]

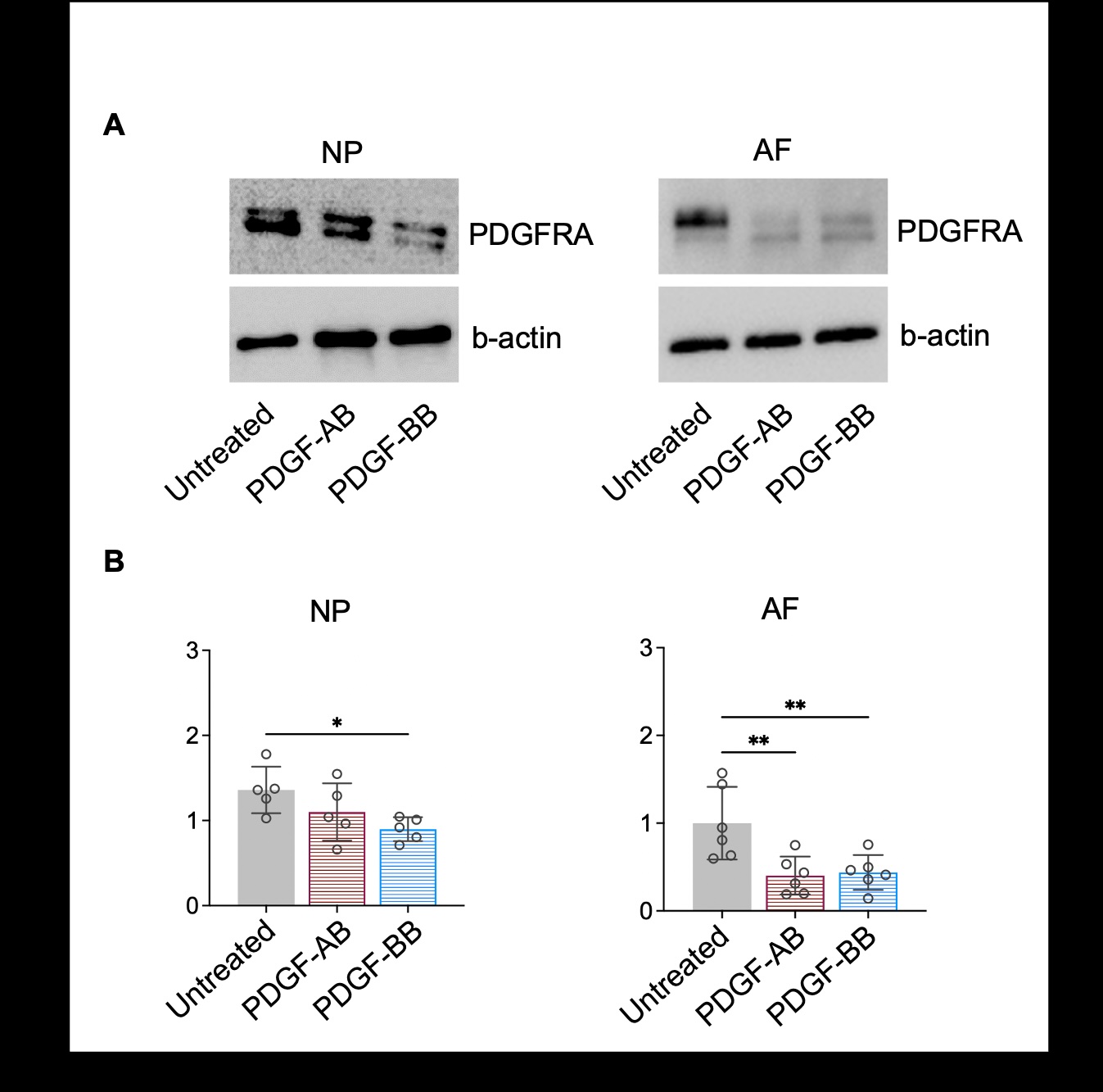

Supplement: Supplement 2 — Supplementary figure 2: Protein expression of PDGFRA was decreased in NP and AF samples treated with rhPDGF-AB and BB. A) Representative images of western blot oof PDGFRA in NP (left) and AF (right) cells. B) Quantification of western blot results showing decreased PDGFRA expression in treated samples compared to the untreated group. NP: n=5; AF: n=6. One-way ANOVA with Dunnett post hoc testing was performed. The data is presented as mean with SD. *p< 0.05. **p< 0.01. [file media-2.jpg]
