# Supplementary material for: Protective effects of PDGF-AB/BB against cellular senescence in human intervertebral disc
Source: bioRxiv. 2024 Oct 13:2024.10.11.617862. Preprint. [Version 1] doi: 10.1101/2024.10.11.617862 (PMC11482872; doi:10.1101/2024.10.11.617862)

bioRxiv preprint doi: https://doi.org/10.1101/2024.10.11.617862; this version posted October 13, 2024. The copyright holder for this preprint (which was not certified by peer review) is the author/funder, who has granted bioRxiv a license to display the preprint in perpetuity. It is made available under aCC-BY 4.0 International license.

Supplementary figure 1: Principle component analysis (PCA) of NP and AF samples treated with rhPDGF-AB and BB. A+B) PCA plot of NP (**A**) cells and AF cell (**B**) showed the distinct cluster between untreated and treated samples. The clusters of PDGF-AB and BB samples were overlapping. NP: n = 5 each group. AF: n = 6 each group.

bioRxiv preprint doi: https://doi.org/10.1101/2024.10.11.617862; this version posted October 13, 2024. The copyright holder for this preprint (which was not certified by peer review) is the author/funder, who has granted bioRxiv a license to display the preprint in perpetuity. It is made available under aCC-BY 4.0 International license.

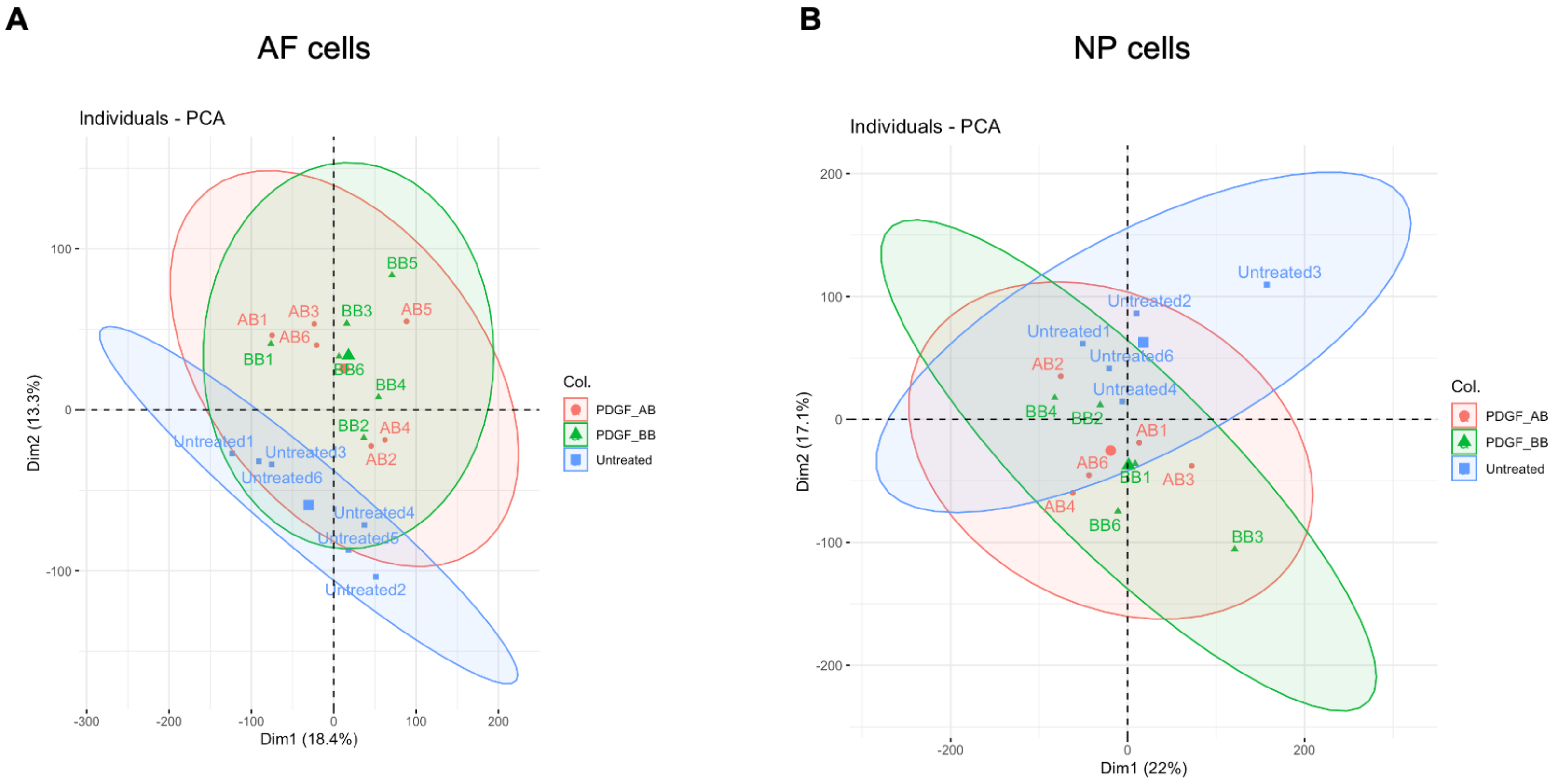

Supplement: Supplementary file 1 [file NIHPP2024.10.11.617862v1-supplement-1.pdf]
